# Detecting Electron Transport of Amino Acids by Using Conductance Measurement

**DOI:** 10.3390/s17040811

**Published:** 2017-04-10

**Authors:** Wei-Qiong Li, Bing Huang, Miao-Ling Huang, Lin-Lu Peng, Ze-Wen Hong, Ju-Fang Zheng, Wen-Bo Chen, Jian-Feng Li, Xiao-Shun Zhou

**Affiliations:** 1 Key Laboratory of the Ministry of Education for Advanced Catalysis Materials, Institute of Physical Chemistry, Zhejiang Normal University, Jinhua 321004, China; cdaqkougotang@163.com (W.-Q.L.); huangbing9158@163.com (B.H.); huangmiaoling886@163.com (M.-L.H.); penglinlu1992@163.com (L.-L.P.); zwhong@zjnu.cn (Z.-W.H.); jfzheng@zjnu.cn (J.-F.Z.); 2 Shanghai Key Laboratory of Materials Protection and Advanced Materials in Electric Power, Shanghai University of Electric Power, Shanghai 200090, China; wenbochen@shiep.edu.cn; 3 State Key Laboratory of Physical Chemistry of Solid Surfaces, Xiamen University, Xiamen 361005, China; li@xmu.edu.cn

**Keywords:** amino acid, single-molecule junction, STM break junction, carboxylic acid, amine

## Abstract

The single molecular conductance of amino acids was measured by a scanning tunneling microscope (STM) break junction. Conductance measurement of alanine gives out two conductance values at 10^−1.85^ G_0_ (1095 nS) and 10^−3.7^ G_0_ (15.5 nS), while similar conductance values are also observed for aspartic acid and glutamic acid, which have one more carboxylic acid group compared with alanine. This may show that the backbone of NH_2_–C–COOH is the primary means of electron transport in the molecular junction of aspartic acid and glutamic acid. However, NH_2_–C–COOH is not the primary means of electron transport in the methionine junction, which may be caused by the strong interaction of the Au–SMe (methyl sulfide) bond for the methionine junction. The current work reveals the important role of the anchoring group in the electron transport in different amino acids junctions.

## 1. Introduction

Understanding the electron transport of biomolecules, including peptides, DNA, and RNA, has received much attention for its relation to daily life activities and potential applications in molecular devices [1,2,3,4,5,6]. Such electron transport has been proved to play an important role in the function of metabolic cycles, enzymatic processes, photosynthesis, DNA damage, and so on [1,3,7,8,9].

Much attention has been paid to the electron transport of peptides, which join electron acceptors and donors with each other and can contribute to the redox reaction between them [3]. Electrochemical methods or scanning tunneling microscopes (STMs) have been used to explore the electron transport properties of peptides, as well as the effect of length, hydrogen bonding, molecular dipole moment, electric field, metal ion binding, and pH on the electrical characterization of peptides [1,3,10,11,12]. Moreover, peptides are formed by amino acid through peptide bonds [2], and the electron transport property of amino acid should have an impact on peptides. Thus, it is also important to study the electron transport of amino acid, which would be helpful in further understanding electron transport behavior in peptides. The tunneling current of amino acid molecules between two electrodes has been measured [13,14], but there is less report on the direct conductance measurement of amino acids junctions [15].

The break junction approach has been demonstrated to be an efficient method to measure the electron transport of molecular junctions [16,17,18,19,20,21]. In this article, we measure the conductance of amino acids by using an STM break junction [17,22,23,24,25,26,27]. Amino acids with different kinds of anchoring groups, including l-alanine, l-methionine, l-aspartic acid, and l-glutamic acid, were measured and compared. Especially, the influence of anchoring groups in electron transport will be discussed.

## 2. Materials and Methods

**Figure 1:**
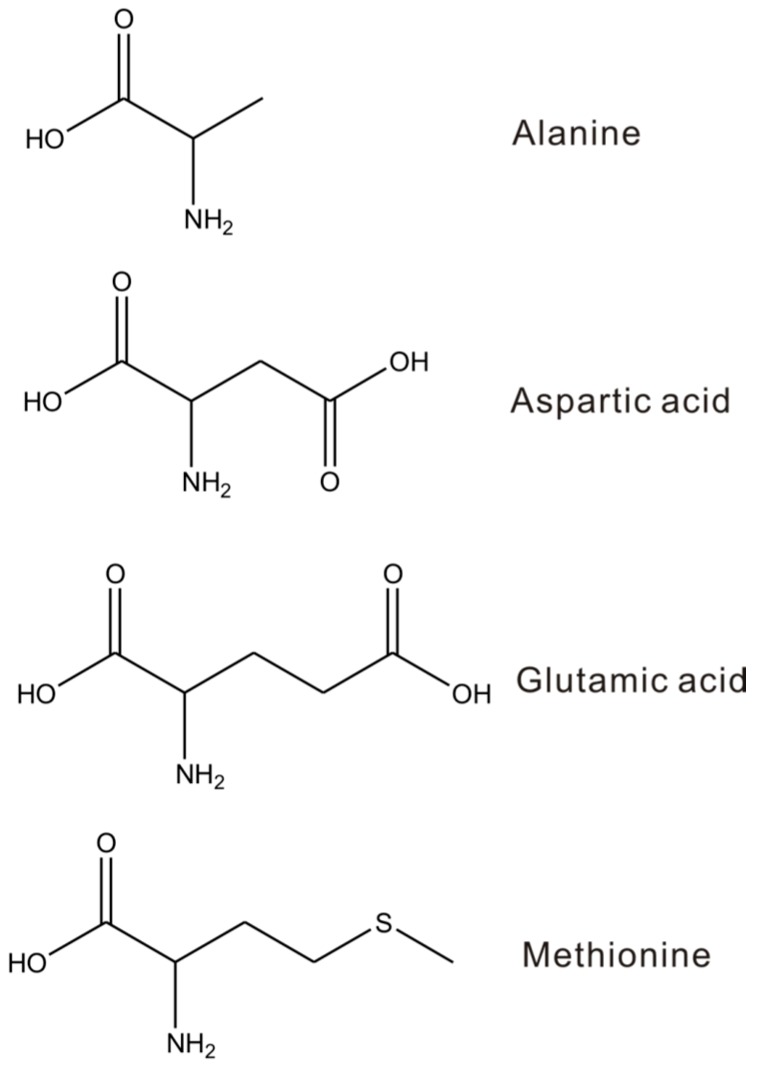
Molecular structures of alanine, aspartic acid, glutamic acid, and methionine.

l-methionine, l-aspartic acid, and l-glutamic acid were purchased from Alfa-Aesar, and l-alanine was purchased from Aladdin. Those molecular structures are shown in Figure 1. Au(111) and mechanically cut Au wire were used as the substrate and tip, respectively. Au(111) electrode was annealed by butane flame before each experiment, which was followed by cooling to room temperature under pure N_2_. The electrode was immersed into an aqueous solution containing 0.1 mM target molecule and washed with ultrapure water (18.2 ΩM cm).

Conductance measurement was carried out using a scanning tunneling microscope break junction (STM-BJ) on the modified Nanoscope IIIa STM (Veeco, Plainview, NY, USA) [28,29]. The STM tip was continually controlled to approach and withdraw the substrate at a constant bias of 100 mV, while the tip current was recorded during the withdrawing progress. The tip withdrawing and current recording speed were 20 nm/s and 20 kHz, respectively. The current curves were treated by the logarithm and binning, and a conductance histogram was constructed. More details can be seen in our previous reports [30,31,32,33].

## 3. Results and Discussion

### 3.1. Amino Acid Only with Anchoring Groups of Amine and Carboxylic Acid

**Figure 2:**
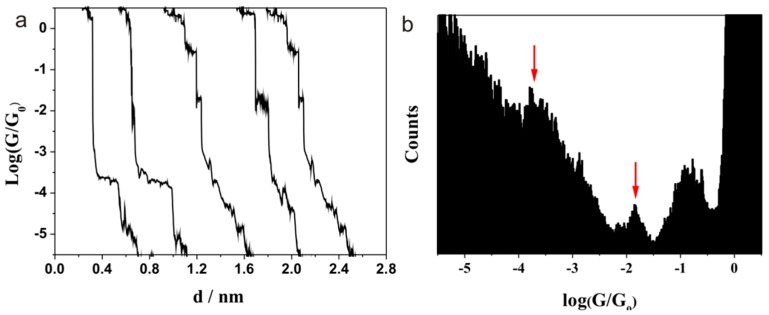
Typical conductance (**a**) curves and (**b**) histogram of alanine (from 1000 curves) contacting with Au electrode at a bias of 100 mV.

We firstly measured the single molecular conductance of alanine, which has only two anchoring groups: amine and carboxylic acid. The conductance value of alanine is shown in Figure 2. Conductance steps around 10^−0.85^ G_0_ (10947 nS), 10^−1.85^ G_0_ (1095 nS), and 10^−3.7^ G_0_ (15.5 nS) can be seen in Figure 2a, while the same values can also been observed in the conductance histogram (Figure 2b). The peak at 10^−0.85^ G_0_ has a much larger conductance value, and such large conductance values can also be found in the literature. This peak might be explained by the conductance of atomic contact influenced by an adsorbed molecule [34,35] or an electrode/π/electrode junction [36,37,38]. Since there is no aromatic ring in the current molecular system, the formation of an electrode/π/electrode junction can be ruled out. Thus, this conductance value can be attributed to the conductance of atomic contact influenced by an adsorbed molecule. The adsorption of O (with Ag) or CO (with Au) can also cause a similar conductance value with respect to Ag [39,40] and Au [41], respectively. However, the exact reason for this peak needs further experiment and calculation. We will not discuss this value since it can be seen in the other systems. The two sets’ conductance values of 10^−1.85^ G_0_ (1095 nS) and 10^−3.7^ G_0_ (15.5 nS) can be attributed to the different contacting configurations between the anchoring group and the electrode [42]. More than 90% of traces show the plateaus, while around 10% of curves have high and low conductance steps in the same conductance trace.

By comparing the conductance values of 2093 nS and 581 nS reported using a conductance screening tool for molecules [15], it may be stated that the different conductance values in the current study may be attributed to the different measurement methods and statistical methods for the histogram. Moreover, the current study was carried out under ambience in air, while an aqueous solution was used for the conductance measurement in the literature [15]. A solvent can also influence the work function of the electrode and change its Fermi energy [43,44], which would alter the conductance value of single molecular junctions. This may cause the difference in the results between the literature and the current study.

### 3.2. Amino Acid with Additional Carboxylic Acid Anchoring Group Besides Amine and Carboxylic Acid

It is also interesting how the electron transport of the molecular junction would change if there were another carboxylic acid in the molecule chain. Thus, the amino acid of aspartic acid and glutamic acid were also studied. Aspartic acid has one more carboxylic acid than alanine, while glutamic acid has one more –CH_2_ unit than aspartic acid. It is expected that these molecular junctions would have many contacting configurations between molecule and electrode, since those molecules have one more carboxylic acid group than does alanine.

**Figure 3:**
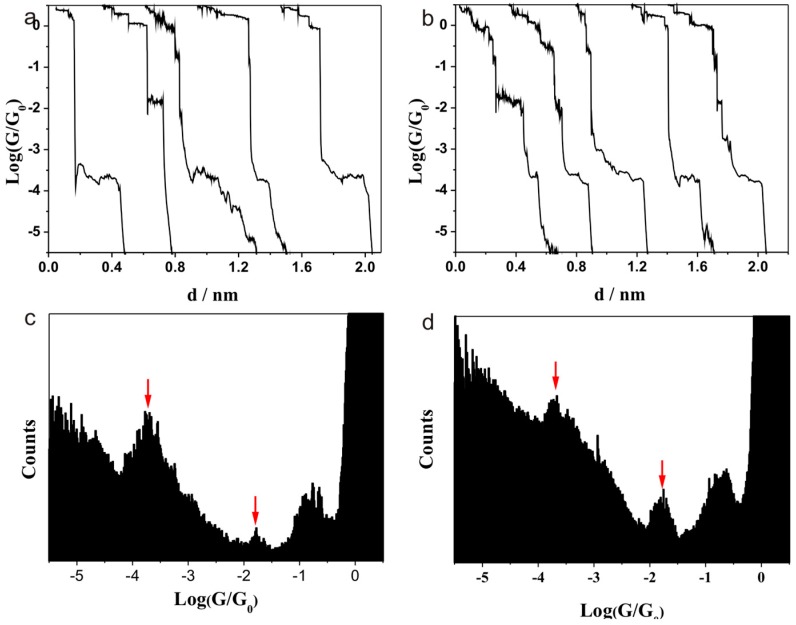
Typical conductance curves for (**a**) aspartic acid and (**b**) glutamic acid. Conductance histograms of (**c**) aspartic acid (from 1380 curves) and (**d**) glutamic acid (from 3000 curves) using Au as electrode.

The conductance measurement of aspartic acid is shown in Figure 3a,c, and conductance values of 10^−1.85^ G_0_ (1095 nS) and 10^−3.7^ G_0_ (15.5 nS) can be found. This shows that the main contacting configuration between aspartic acid and Au is the same as that for alanine and Au, since similar conductance histograms are found for alanine and aspartic acid. In other words, the aspartic acid junction should have the same electron transport as that of alanine junction, and the backbone of NH_2_–C–COOH, not NH_2_–C–C–COOH, is the primary means of electron transport for aspartic acid–Au junctions.

We also carried out an experiment on glutamic acid. Interestingly, 10^−1.85^ G_0_ (1095 nS) and 10^−3.7^ G_0_ (15.5 nS) were also found for this amino acid (Figure 3b,d). This result further demonstrates that such amino acids contact the electrode through the backbone of NH_2_–C–COOH. The configuration of the two carboxylic acid groups binding to both ends of the electrode is not favored. The chain of NH_2_–C–COOH is the main configuration for the formation of junctions with aspartic acid and glutamic acid.

### 3.3. Amino Acid with Methyl Sulfide (–SMe) Anchoring Groups Besides Amine and Carboxylic Acid

Now, we focus on the amino acid with additional methyl sulfide linker to explore the binding site of the molecular junction. Methionine was chosen for its methyl sulfide group, which can bind to the Au electrode [45].

**Figure 4:**
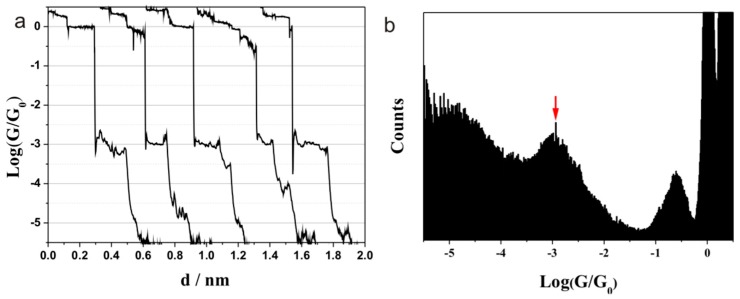
Typical conductance (**a**) curves and (**b**) histogram of methionine (from 1150 curves) contacting with Au electrode.

A conductance value at 10^−3^ G_0_ (77.5 nS) was found for methionine, while no peaks at 10^−1.85^ G_0_ (1095 nS) and 10^−3.7^ G_0_ (15.5 nS) were observed (Figure 4). These conductance values together with other amino acids are summarized in Table 1. The difference between methionine and other amino acids may be caused by the strong interaction of Au–(SMe) in methionine. The breaking force for Au–(SMe) is 0.7 nN, while 0.6 nN is found for Au–(NH_2_) and Au–(COOH) [46,47], which shows that the Au–(SMe) bond is stronger than the other two. Comparing the different breaking forces among Au–(SMe), Au–(NH_2_), and Au–(COOH), it is favorable to form Au–(SMe) contact during the self-assembly monolayer. Thus, the formation of single molecular junctions may be between Au–(SMe) and Au–(NH_2_) or between Au–(SMe) and Au–(COOH), and fewer junctions are formed between Au–amine and Au–carboxylic acid. The current result with no conductance peak at 10^−1.85^ G_0_ (1095 nS) or at 10^−3.7^ G_0_ (15.5 nS) also supports this suspecting. Furthermore, the conductance of methionine (77.5 nS) is lower than the high value of alanine, aspartic acid, and glutamic acid (1095 ns). This may be caused by the longer electron transport (MeS–C–C–NH_2_ or MeS–C–C–C–COOH) for methionine compared to that for the other amino acid (NH_2_–C–COOH).

## 4. Conclusions

We have measured the single molecular conductance of amino acids, including alanine, methionine, aspartic acid, and glutamic acid. The results show that those amine acids with only anchoring groups of amine and carboxylic acid bind to the electrode through the backbone of NH_2_–C–COOH. For a strong interaction between Au–(SMe) bond, NH_2_–C–COOH is not the primary means of electron transport in the methionine junction. The current work shows the important role of the anchoring group in electron transport in the amino acid junctions.

## Figures and Tables

**Table 1:** Summary of single molecular conductance of alanine, aspartic acid, glutamic acid, and methionine.

Molecules	Conductance (nS)
Alanine	1095, 15.5
Aspartic acid	1095, 15.5
Glutamic acid	1095, 15.5
Methionine	77.5
